# When the digits don’t add up: Research strategies for post-digital peacebuilding

**DOI:** 10.1177/00108367231184727

**Published:** 2023-08-12

**Authors:** Andreas T Hirblinger

**Keywords:** digitalisation, digital technology, peacebuilding, post-digital, social media

## Abstract

This article develops a post-digital perspective for the study of international peacebuilding and elaborates its merits. Contrary to narratives in policy and practice that tend to fetishize the digital, digital peacebuilding cannot be meaningfully separated from peacebuilding before digitalization. Resisting the call for a “digital turn,” a post-digital lens helps to research, rewrite, and rework the digital while simultaneously staying with and moving beyond digitalization. It aims to demystify the role of digital technologies while enabling critical scrutiny of their impact on contemporary and future peacebuilding. More specifically, the post-digital helps us to (1) establish a critical distance to narratives of fast-paced innovation and progress that fetishize the digital, (2) scrutinize how digitalization compounds contemporary approaches and constellations of peacebuilding, (3) engage with the uneven temporalities of digital peacebuilding and its diverse global manifestations, (4) shed light on its real, embodied, and tangible effects on conflict-affected populations, (5) hold digitalization accountable by unearthing disillusionments and failures, (6) re-adjust our focus on human agency in the development and use of the socio-technical systems that constitute digital peacebuilding, (7) and finally, take a rhizomatic view that is concerned with how power relations make and break digitalized peacebuilding networks.

There has been an increasing integration of digital technologies into international peacebuilding efforts over the past decades, and with it, a heightened interest in digital peacebuilding as a research topic. Digital technologies are now discussed in terms of their contribution to conflict analysis, early warning, ceasefire monitoring, the protection of civilians, peace mediation, and reconciliation, among others. We could describe this development as a *turn* toward digital peacebuilding caused by a combination of technological innovation, accelerating policy and practice needs, and a growing research interest stemming *inter alia* from the realization that conflict-affected contexts are increasingly digitized (Sandvik et al., 2014), a trend toward remotely executable interventions (Perera, 2017), or hopes that technologies may provide new opportunities for “bottom-up” peacebuilding (Tellidis and Kappler, 2016). The turn toward the digital in peacebuilding is performed in policy- and practice-oriented contributions that equate the term “digital technologies” with “new technologies” (Wählisch, 2020), mobilize for a “data revolution” (Mack 2014) (Independent Expert Advisory Group, 2014), and aim to guide the “digital transformation” of the sector (United Nations Peacekeeping, 2011). Moreover, the emerging academic scholarship on the topic invokes similar notions when referring to a “digital revolution” (Firchow et al., 2017: 5), the possibility of a “digital renewal” (Richmond and Tellidis, 2020), or indeed a “potential digital turn in peace and conflict studies” made necessary by a “shift from ‘analogue’ to ‘digital’ approaches in international relations” that affects the “international peacebuilding architecture” (Richmond et al., 2023).

Yet, when attempting to get to the bottom of such sentiments, we find that no clear line can be drawn between an assumed pre-digital era and the contemporary condition. Today’s “digital revolution” is led by technologies such as machine-learning, remote satellite systems, and virtual reality. Yet, calculators, radios, and telephones that were essential peacebuilding tools for decades likewise operated through the basic elements of digital technology: discrete and countable units used to store, transmit, or reproduce information (Cramer, 2015: 15–16). In terms of the temporal dimension of digitalization, it may thus be more fitting to think about the long durée and the gradual, generational change toward increasingly—but never completely—digitized peacebuilding approaches (Schirch, 2020). In terms of the spatial dimension of digitalization, we must also be cognizant of the limits of the digital, including concerns about a global “digital gap” in infrastructures, access, and literacy between the global North and South and between different demographics (Njeru, 2009). However, a stark differentiation between worlds that have turned to the digital versus worlds that have not (yet) done so rests on binary thinking that creates otherness. While this is an understandable result of modernist thought (Goga, 2015), analytically, it is of little help. We do not live, fight, and make peace digitally in one world and analogically in the other because dynamics of peace and conflict traverse digital and non-digital spaces and times.

When it comes to their effects on contemporary peacebuilding, the presence of the digital somewhere cannot be thought of without the absence of the digital elsewhere. Where digital peacebuilding initiatives aim to involve populations with limited digital access or literacy in places such as Somalia or Burkina Faso, including through digital capacity development, infrastructural interventions, and the use of “low-tech” applications such as WhatsApp (Meier, 2021), these efforts are inextricably linked to the global centers of digitalization, where digital technologies and digital peacebuilding approaches are developed and devised. Moreover, while they will extend opportunities for digital participation to some parts of the population, they will in the same movement exclude people without digital access who would otherwise join through non-digital means of participation. In other words, contemporary peacebuilding unavoidably cuts across digital and non-digital dimensions. These dynamics unfold in a global trajectory of economic, political and technical integration, shaped by heterogeneous and often contradictory experiences and narratives of digital (post)-modernity and (de)-colonialism. (Leander, 2021; O’Hara, 2020). In short, when seeking out opportunities for transformative change toward a more peaceful world, the digital and the non-digital can’t be thought of as a binary, only as a dyad in which both elements matter in relation to each other.

All this suggests that there may be no digital turn to speak of. Declaring a turn tends to enhance scholars’ position and build their social capital in the field (Baele and Bettiza, 2021). However, the invocation of a digital turn in peacebuilding mystifies rather than clarifies the role of technology in peacebuilding. As a topic of research, the digitalization of peacebuilding constitutes a problem that is *ontological* as much as *methodological*: it poses questions about what contemporary and future peacebuilding is made of, how we should conceptualize it, and how we can study it meaningfully (Jackson, 2011; Klotz, 2007). The emerging scholarship on this topic has grappled with these concerns to some extent, yet it seems worthwhile to take stock of this discussion—and move it forward—which is what this article intends to do. It aims to formulate suggestions for how researchers can engage with the effects of digitalization without falling into conceptual tunnel vision that, while enabling stark claims, may do injustice to the messy and relational nature of peacebuilding and peace research (Perera, 2017; Shinko, 2008) and ultimately disfavor those affected by armed conflict.

Most, if not all, researchers who have engaged with this topic would likely agree that technology and digitalization are not a panacea for peacebuilding—nor will they blame technology for all its failures. Therefore, engaging with the digital in peacebuilding requires a research strategy that simultaneously enables us to turn toward and away from the digital. I suggest that this may be achieved through engaging with the notion of the post-digital and operationalizing it as a research perspective. We have learned from past turns in peacebuilding research that they come with the risk of ignoring developments in other disciplines and investing too little into the exchange of ideas, tools, and concepts with them (Schierenbeck, 2015), thus likely repeating intellectual mistakes that have already been grappled with elsewhere. Therefore, this article draws on explorations of the post-digital in other sub-fields of the humanities and social sciences (Jandrić et al., 2018, 2019; Sinclair and Hayes, 2019; for an overview, see Taffel, 2016). Notably, the post-prefix should not be understood as an attempt to move to a time after digitalization and create a sense of (temporal) distinctness but as an attempt to obtain a reflexive position vis-à-vis the rhetorically and performatively constructed appeal of digitalization. The term invites us to rewrite and rework the digital by staying with what comes after digitalization—understood not in terms of linear, temporal progress, but in terms of a concern with the impact of digital technologies on the world (Peters and Besley, 2019; Sinclair and Hayes, 2019).

Lessons from past turns in peacebuilding also suggest that they often fail to relate research to practice- and policy narratives, with which scholars will necessarily have to engage critically if they want to contribute to change (Hunt, 2023). Therefore, the article discusses the elements of a post-digital research perspective against the backdrop of an analysis of social media posts that construct a relationship between the digital and peacebuilding. Given that Twitter is used mainly for professional purposes and the condensed content of Tweets, I suggest treating the archive of social media posts as a concentrate of public discourse that, while not containing all details and nuance, can help identify many of the most critical claims and assumptions that inform digital peacebuilding policy and practice (compare to Sam, 2019).^1^ Without a doubt, the practice discourse reconstructed with this approach is highly exclusive, primarily containing the narratives of an educated and well-resourced social media sub-population that shares content to engage professionally with digital peacebuilding, for instance, to promote reports, guidance, projects, or events and trainings. It says comparatively little about how ordinary populations—including those affected by conflict—may think about the digital. Nonetheless (if not therefore), the archive contains a relevant subset of the policy and practice discourse to which academic research may want to speak back.

My discussion reflects on the tropes of digital peacebuilding visible in the Twitter archive against the backdrop of a larger narrative framework concerned with the impact of digitalization on society and politics. It makes suggestions for how researchers can critically engage with these tropes through seven^2^ distinct intellectual maneuvers. Compared to turns that are often aimed at provoking a radical change in the academic debate that hardly ever translates into impactful critique, these maneuvers are meant to provide a repertoire of thought processes that may help us navigate the complexities of digitalization in ways that are more open to the messiness of peacebuilding. Rather than providing a neat theoretical framework, they are meant to sketch out a research perspective that can help us to simultaneously stay with, and move beyond, the digital. They invite us to seek critical distance from narratives of fast-paced and homogeneous change and instead move toward a concern with how digitalization produces a heterogeneity of adverse impacts in institutional, material, and embodied manifestations that ultimately remain the responsibility of us humans. As we open up to the post-digital, peacebuilding emerges as rhizomatic dynamics of socio-technical relations that are constantly rewoven—ranging from institutional partnerships and linked infrastructures, to individual human–machine relations and entanglements of data, neural networks, and human thought. Digital peacebuilding develops its character and outcomes by making connections between *some* social and technical elements and breaking relations with others—determining how peacebuilding is done and with what effects. This is where the critical potential of the post-digital becomes most pertinent: it encourages the critical scrutiny of these dynamics and their impact on contemporary and future peacebuilding.

## De-accelerating digital peacebuilding

Among many peacebuilding practitioners and policymakers, the digital is strongly linked with notions of “transformation” and “change.” This is visible in references to a digital “era,” “age,” or “revolution,” characterized by pressing challenges that require distinct innovations that are “emerging” or “evolving.”^3^ Yet, the sense of fast-paced digitalization, which requires a digital peacebuilding response, is anything but new. The first uses of information and communication technologies (ICTs) date back to the early 2000s, when global initiatives such as ICT4Peace were created with the aim of harnessing the “extraordinary power of information and communication technologies” for peace and security by highlighting the uses of ICTs to promote peace and establishing a “framework” and “good practice” that would create “new opportunities for innovation and growth” (Stauffacher et al., 2005: 7, 52). About a decade later, a second wave of initiatives unfolded around the term “PeaceTech” (Dajer, 2018), driving the idea that “technological innovation offers promising approaches to the development of more effective strategies for conflict prevention and peacebuilding,” as Helena Puig Larrauri and Yeonju Jung (2017) put it. At the multilateral level, the UN Secretary General’s Initiative on New Technologies similarly stressed their instrumental nature for the implementation of the UN Charter and UN mission mandates and pledged to keep up with the scale and speed of innovation (United Nations, 2018). The impetus to innovate also became increasingly institutionalized through “accelerator grants” and “peace-tech” labs that promise to leverage the benefits of the latest technologies for peacebuilding and conflict prevention (see for instance, PeaceTech Accelerator, 2017).

Yet, we may want to be skeptical about modernist framings that portray digital peacebuilding as both temporally distinct and progressive. To this end, a post-digital perspective invites us to obtain a critical distance from the obsession with technological innovation as an isolable phenomenon. Proponents of the post-digital have often critically engaged with Nicholas Negroponte’s (1998) famous proclamation that “the digital revolution is over.” Comparing digital technologies to earlier technological innovations such as plastic, early discussions of the post-digital argued that digital technology neither formed an integral nor sufficient building block for innovation and change. Such portrayal was built on the premise, which is contained in notions such as “digital turn” or “digital revolution,” that the digital once emerged in distinction, or separation, from a non-digital world. However, the notion of a time before and after digitalization has been dismissed by a large corpus of studies pointing to the immanent materialism of digital technologies, which all point to how the “real” and the “virtual” have “always coexisted within a single plane of material reality” (Taffel, 2016: 5). Nonetheless, the post-prefix helps to deconstruct the temporal and ontological distinctness of the digital and to acknowledge the normalcy of the digital condition. While tech optimists may use this insight as a starting point to engage in more fine-grained discussions of the potential of the latest innovations, such as quantum computing (Halpern, 2019), it also provides an important starting point for more critical perspectives. Taffel suggests that the post-digital may help us to “abandon the fetishization of the new (. . .) present in calls to move to the next big thing” (Taffel, 2016: 7). The post-digital thus encourages us to literally “step out of the accelerator” that causes a tunnel vision obsessed with progress, while reducing our focus on the context.

This does not mean that research on digital peacebuilding should simply ignore progress or innovation altogether. Rather, it means that instead of taking claims about progress at face value and focusing our research on the latest innovation, we may study how technological progress is performatively enacted across the peacebuilding sector and with what effects. For instance, we may want to explore how notions of progress constructed by digital peacebuilding initiatives create their own post(-)realities, thus performatively outdating established approaches and potentially replacing actors and practices. For instance, PeaceTech networks or start-ups increasingly acquire project funding and consultancy contracts from internal donors and use these resources to advance methodologically different approaches to conflict analysis, public consultation, and dialogue that harness big data and artificial intelligence (AI).^4^ These innovations are usually driven by good intentions– such as making peacebuilding efforts more efficient, inclusive, or evidence-based, but they will also compete with, and potentially replace, established peacebuilding approaches and practices. Rather than progress, this could lead to regress. For instance, as Mark Duffield (2016) has prominently argued, digitally-enabled remote governance made engagements with conflict-affected populations quicker, more precise, and less risk-prone, but it also led to a “resilience of the ruins,” as transformative change remains elusive. To de-accelerate digital peacebuilding thus means to resist blindly bandwagoning around tales of progress. As we accept and normalize the digital condition, our research will be able to move behind the lures of innovation and change to study its effects in the here and now.

## Composting digital peacebuilding

Many references to the digital in the Tweets analyzed for this article also invoke its ability to provide “solutions” to peacebuilding problems and to enable “tech-driven” peacebuilding through “data,” “tools,” or “apps.” In contrast, very few tweets point to their possible limitations, for instance, in the context of reconciliation efforts or meaningful dialogue. Countering such tech-solutionist narratives, the notion of the post-digital invites us to express a “disenchantment” with the digital (Cramer, 2015: 12). In popular culture, such repositioning may express itself in metaphorically “switching off” the digital, encapsulated in memes or artwork that play with the imaginary return to what are perceived as pre-digital or analog techniques and practices. However, in practice discourses on digital peacebuilding, there are comparably few references to continuing or returning to “offline” and “analog” forms of peacebuilding.^5^ A post-digital perspective comes with the realization that it is impossible to obtain complete freedom from the effects of digitalization, as every move beyond the digital will necessarily happen on the “canvas” of the digital (White, 2009). This means that the move *beyond* requires simultaneously staying *with* it through a concern with how past and present manifestations of digitalization affect the present and future of peacebuilding. Unsurprisingly, explorations of the post-digital have benefited from drawing parallels to debates around post-colonial and post-humanist thought, which stress the importance of investigating the historical trajectories that continue to yield their influence in the present and the future in their discursive, performative, and material dimensions (Sinclair and Hayes, 2019: 126).

This suggests we may want to be concerned less with what happens next and more with what has already happened and how that conditions present and future peacebuilding. Negroponte’s (1998) well-aged claim that “the connotation [of the digital] will become tomorrow’s commercial and cultural compost of ideas” can support this intellectual maneuver. The notion of “compost,” re-appraised critically through the reflections of Donna Haraway (Franklin, 2017; Sinclair and Hayes, 2019), invites us to think of the post-digital not as something that comes *after* the digital, or to declare digitalization and its effects as *passé*, but to investigate digitalization as a breeding ground for new approaches, practices and constellations of peacebuilding. And as we are concerned with compost, we may want to ask whether this process produces a fertile ground for peace or just a dumpsite on which conflict continues to fester. Curiously, the first recorded tweet mentioning “digital” and “peacebuilding,” by Sanjana Hattotuwa, a pioneer of the ICT4Peace community, linked to a blog post entitled “Inside the Digital Dump”. The post featured a picture of digital waste taken from a photo essay in the Foreign Policy magazine and included the message “technology drives the forces of globalization,” thus suggesting that the digital, even in its decommissioned or recycled manifestations, is linked to social inequality and violent conflict. That said, when “composting” the digital in peacebuilding, we may want to literally investigate the various components that digitalization offers to peacebuilding as a field of practice and ask about what grows from their mélange. It is important to remember that successful composting requires the right balance of various components—and that not all may be suitable for peacebuilding.

For instance, current digital peacebuilding initiatives put great effort into enhancing data- and evidence-based approaches that enable better empirical insights for conflict prevention as well as for the planning, management, and evaluation of initiatives (Panic, 2020). The current decade has also seen an increasing introduction of monitoring capabilities for peacekeeping, be it through satellite-based earth observation or social media analytics platforms (Karlsrud, 2014; Mac Ginty, 2017). Yet, while such technological innovation seems to enhance the intelligence capabilities of third parties, we notice a relative lack of efforts to use technology in ways that can facilitate meaningful encounters between conflict parties that would enable dealing with grievances and past injustices. For instance, during virtual meetings or consultations peacemakers may have a “missing sense of peace” due to a lack of in-person interaction (Bramsen and Hagemann, 2021). Composting as research practice thus means accounting for the various digital components that are offered to peacebuilding—including the ideas we and others have about them—and asking about their effects on how peacebuilding is conceived and practiced. This is not to say that there is one correct balance of digital components (as is the case for organic compost) that provides a fertile ground for peacebuilding. Indeed, we may want to metaphorically stir that compost around to identify disbalances that make certain organisms grow but not others. What, for instance, explains the relative dominance of remote sensing, data analytics, and surveillance technologies in peacekeeping? For example, does technological innovation in this field benefit disproportionately from the proximity of this practice field to the military and associated private firms that push innovation? And are social media data analytics advancing because the same technologies can be used for profit-driven market research? The following sections will provide further suggestions on how to answer these and similar questions.

## Molecularizing digital peacebuilding

Accepting digitalization as the new normal does not mean assuming that its manifestations are globally homogeneous. While investigating practices through which technological progress was and is enacted performatively and discursively, we must acknowledge the globally uneven nature of these dynamics. The practice discourse at times acknowledges challenges stemming from the “digital divide,” and it strongly highlights the need for “inclusive” or “participatory” uses of technology, mainly to involve “women” and “youth.” However, peacebuilding practice nonetheless seems to think of digital approaches in a universalizing and homogenizing manner—something that simply requires “equality” and “access.”^6^ Such digital universalism is also echoed in scholarly discourses primarily speaking from the vantage point of late industrial societies. Discussing the post-digital, some scholars similarly aim to capture the essence of the post-digital by spelling out what they consider to be its most salient features. For instance, Peters and Besley (2019) use the post-liberal call for a seemingly universal “critique of digital reason” composed of two main elements, namely a concern with “mathematico-technical control systems” and their political economy. Similar approaches are emerging in peacebuilding studies, for instance, when Richmond and Tellidis (2020) introduce the notion of “digital governmentality” to argue that digital innovation will likely perpetuate top-down peacebuilding approaches, thus risking to leave concerns with the globally heterogeneous nature of digital peacebuilding aside. On the contrary, they—and others—have also demonstrated a concern with Eurocentrism, domination and “Western hegemonic order” (Richmond et al., 2023: 28).

At the same time, the practice discourse is disproportionately concerned with providing digital “solutions” to places other than those that lead global digital innovation: on the African continent and in countries such as Iraq, Myanmar, or Sri Lanka. As such, they reproduce a Eurocentrism that simultaneously declares Western or European technological systems as points of reference while projecting them to the Global South. We may want to ask if the notions of a globally homogeneous digitalization are more a result of a concern with liberal rhetoric promoting universal access and connectivity to open new technology markets than of the actual dynamics of digitalization that play out in a much more heterogeneous fashion—both globally and domestically (Dutton and Reisdorf, 2019; Gallagher and Knox, 2019). While a concern with the power of Western stares and US-based Big Tech is certainly warranted, it is important to ask how far this power indeed reaches—and what happens to digitalization in those conflict-affected contexts where it is rather limited—such as Syria or Iran. A post-digital approach may motivate us to ask further about the uneven temporalities through which the emergence of such arrangements is characterized, for instance, by reflecting critically on the notion of the digital divide. As Jandrić et al. (2019: 166) put it, “to be on the ‘worse end’ of the ‘digital divide’ does not mean that you live an entirely ‘analogue’ life, unaffected by the encroachments of digitization” but that you may “have less agency” and “that you are undoubtedly impacted to a greater extent by a technology.” More importantly, we may rather speak of an “ongoing series of molecular digital revolutions that continue to enact changes in heterogenous ways across different geographies” (Taffel, 2016: 5). For instance, in places such as South Sudan or Mali, where Internet penetration is relatively low, social media hate speech and misinformation are nonetheless rampant and powerful. And they may be the consequence of the relative absence—not presence—of the public and private sector’s governmental intervention in the digital space (Defy Hate Now and Center for Strategic and Policy Studies, 2021).

Limited digital access or literacy may also mix in more peculiar ways with established peacebuilding approaches and doctrines, creating processes and relations that are not just simply less digital but just different. Efforts to apply “human-centered” design and “localized” technological approaches in areas with limited technical infrastructure stem from a trend to circumvent global inequalities in digitalization, for instance, via the deployment of technologies that require little data and little digital literacy. Practitioners choose to employ such technological innovations based on their judgment of the readiness of specific conflict-affected contexts to avoid adverse effects, notably excluding the digital have-nots. For instance, UN agencies have employed WhatsApp to facilitate online consultations in countries such as Somalia or Burkina Faso (Meier, 2021), while text-message-based systems are widely employed in crowdsourcing applications such as Ushahidi that emerged in Kenya (Kahl et al., 2012). Yet, while aiming at human-centric or localized design, peacebuilding actors also reproduce and normalize the globally uneven and often hierarchical landscape of digital peacebuilding. Digital peacebuilding initiatives tend to adjust their approaches to these uneven temporalities in digitalization by, for instance, promoting low-data solutions and simple tools such as WhatsApp for participatory processes. The de-facto spread of digital innovation in peacebuilding is thus globally heterogeneous, with more advanced technologies operated by skilled analysts in data centers located in the global North, coupled with simpler and less-demanding technologies employed in the conflict context often found in the Global South. Therefore, our research should molecularize digital peacebuilding through a curiosity toward such digital practices that do not only differ in the degree of digitalization (i.e. less bandwidth) but also in its quality (i.e. in terms of distinctly different approaches to peacebuilding).

## Grounding digital peacebuilding

Most digital peacebuilding interventions are devised to discretely target what is understood as a distinct “space,” portrayed as “digital,” “cyber” or virtual,” and “online,” in contrast to “offline.” They are mainly concerned with aspects of “media,” “communication” and with responses to conflict that are of a behavioral nature, such as “campaigning,” “activism,” “education,” “diplomacy,” “storytelling” or “games.” Likewise, the phenomena that they aim to address are also largely viewed as being in—and the result of—the digital sphere, such as “polarization,” “hate speech,” or “disinformation.” The digital itself is commonly viewed as a “driver” of conflict, while concerns with social, political, and economic “causes” of conflict, such as “poverty,” play a marginal role.^7^ However, a post-digital lens rejects the conceptual shift implied in the digital revolution toward an exclusive focus on the virtual or the digital (Pepperell and Punt, 2000 cit. in. Taffel, 2016), and it remains critical of a view of the world as reducible to a discrete set of computational problems (Morozov, cit. in Jandrić et al., 2018).

Digital technologies blend with the lifeworld of those who live in conflict and those who aim to prevent it. While usually still contained in “hardware,” these technologies interact with human bodies in increasingly ubiquitous manners, defining how we go about our everyday professional life in ways that blur the boundaries between humans and machines (Clayton et al., 2015). Far from being merely *virtual*, these socio-technical relationships have distinct material and embodied dimensions—being built and maintained with the help of conflict-financing rare-earth elements (Brennan, 2017) while promising to protect humans from physical harm through alerts distributed via smartphone applications (Kahl et al., 2012). Rather than approach digital peacebuilding as something that happens in separation from the “real,” as the association of the digital with the “virtual” may suggest, the post-digital invites us to engage with its very tangible effects. It asks us to reckon with the fact that “the cyberspace has insidiously insinuated itself into our existence, at every scale and every turn,” which makes it impossible to keep up a binary opposition between the “digital” world and the “normal,” “real” world (Spiller, 2009: 95–96). Ryberg (2019: 165) described the post-digital as being about “dragging digitalization and the digital—kicking and screaming—down from its discursive celestial, ethereal home and into the mud.” As he suggests, “it is about rubbing its nose in the complexities of everyday practice” because digital technologies are implemented in “messy, political, social and organizational contexts that are constantly changing and that will shape, and will be shaped by ‘digitalization” (Ryberg, 2019: 166).

Transferring this stance to the study of peacebuilding, we can ground digital peacebuilding by asking about how digitalization not only shapes but is shaped by the political, social, and organizational contexts that constitute peacebuilding. While acknowledging that in today’s world, the digital is stitched into almost every aspect of reality, we should move beyond an opaque notion of such entanglements by asking about the institutional, embodied, or material dimensions of building peace with digital technologies. This first entails shedding light on the discursive and performative construction of digital peacebuilding as a separate field of action, for instance, through a shift in the discourses and practices of specialized non-governmental organizations (NGOs) and international organizations. Furthermore, we should investigate the effects of its composite nature, including its anchoring in international politics, international bureaucracies, and the market. For instance, several organizations have established dedicated expert positions, working groups, or training courses to mainstream digital approaches in the sector—increasingly attracting financial resources and supplying new skills, knowledge, and approaches that shape the work of a new generation of peacebuilding professionals. Specialized digital methods and applications may also further stimulate a consulting and outsourcing approach to peacebuilding, as they can be readily packaged and instantly sold as “tools” by small and agile tech labs and start-ups, such as through subscription and advisory services. However, the digital marketization of peacebuilding may further fragment the field and make sustainable interventions and systemic long-term change more difficult. Nonetheless, a trend toward digital approaches is also visible in the strategic priorities of prominent peacebuilding actors, with the promotion of digital skills and technologies increasingly becoming a priority of institutions such as the UN Peacebuilding Commission and the UN Peacebuilding Fund’s 2020–2024 strategy focusing on countering hate speech (cf. United Nations Peacebuilding, 2020). As in other fields of society, investments in digitalization will naturally mean that other approaches will receive less attention and funding.

Grounding digital peacebuilding also requires being sensitive to the material effects and implications of digital approaches on conflict-affected populations, as well as studying the connects and disconnects between a digitally constructed peace, the visceral and embodied experiences of conflict-affected populations, and their material living conditions. For example, efforts to fight hate speech are commonly justified through the claim that social media platforms provide a breeding ground for the spread of narratives, opinions, and misinformation that results in tangible violence. Yet, we should be equally concerned with the social, political, and material factors that drive online hate speech and interrogate to which degree digital responses are equipped to respond to them (Denti and Faggian, 2021). That said, we should no longer (or not only) look at a “virtual” peace as something that is merely represented or constructed through digital media. It means considering fake news and disinformation not solely as “wrongful representations” of reality but engaging with their very real, embodied effects in terms of hatred and violence, as well as in terms of the material, organizational and systemic factors that enable these digital realities to shape both war- and peacemaking.

## Summoning digital peacebuilding

Most of the practice discussion on the role of digital technologies in peacebuilding is centered on how they may provide particular “solutions” and, to some degree, on exploring their “potential,” “promise,” or “benefits.”^8^ However, the notion of the post-digital encourages a look beyond the instrumental value associated with digital technologies to explore critically what influence these technologies exercise, as well as the “unease, fatigue and disillusionment” that come with it (Berry and Dieter, 2015; Jandrić et al., 2018, 2019). Translating this attitude to the study of peacebuilding, we can summon the digital—by scrutinizing its promises and limits not in separation from, but in relation to, the social and political context in which technologies are brought to bear. The research and practice of digital peacebuilding has almost exclusively been concerned with digital technologies as drivers of conflict and tools for building peace. In contrast, comparably little has been said about how digitalization may negatively affect peacebuilding practice. What is lacking is an engagement with the challenges and disillusionment that emerge from the utilization of digital technologies in peacebuilding.

Summoning the digital is not merely an intellectual or academic task but a practical one as well. It can take the form of resistance to mark the detrimental effects of digitalization. As in other fields (Andrews, 2002), the use of digital technologies in peacebuilding often creates a digitally polished aesthetics absent of social and emboddied experiences, such as spending hours on uncomfortable chairs in unacclimatized makeshift workshop venues or facing physical insecurity when traveling to a consultation or negotiation. While convenient at first, the absence of such experiences may have adverse effects on peacebuilding. Those who have taken risks, traveled far and endured the discomfort of night-long negotiations may be more willing to make peace than those who can easily end a meeting with a mouse click. Staged online workshops, where microphones can be muted, and exposure to information can be carefully controlled, leave participants with fewer cognitive and emotional experiences, and reduce opportunities for spontaneous and honest encounters. Research can explore such glitches of digitalization by querying the disenchantment that those who build peace encounter when using digital technologies and by investigating the practical responses to them. We may also hold the digital accountable by unearthing the disconnects between assumed technological and social progress and peacebuilding practices and outcomes specifically.

However, since the lines between what is digital and what is not can no longer be clearly articulated, we must be cognizant of our inability to sufficiently separate the impact of technological artifacts from their social embeddings. As Arnold and Pearce (2008: 49) have argued, if we assume that humans and nonhuman technologies are jointly implicated in the “causal chain that had led to a bad outcome, the cast is so huge, the guilt spread so thin, that there is scarcely any point in attributing accountability or responsibility at all.” Nonetheless, we may be able to determine the impact of a particular technological artifact, for instance, by asking about the implication of technology in specific causal mechanisms and by asking what difference technology made for a particular outcome. For example, we may differentiate between the initial outputs produced by a technology (such as an AI model) and the outcomes that result from the interaction between these technology outputs and human decisions and actions (Busuioc, 2021: 828). More concretely, machine-learning approaches may promise to enable fast, efficient, and widespread participation in peace processes through the real-time collection and analysis of large amounts of natural language inputs—for instance, in the context of online focus groups (Bilich et al., 2019). Such methods may prove handy for mediators or decision-makers who wish to obtain a “representative” picture of a population’s preferences. However, we know little about how such remote efforts compare to in-person consultations or town hall meetings that may generate public support and build the legitimacy of processes by providing the participants with a visceral experience of being heard.

Summoning the digital must go further than merely establishing the unintended effects of technology. It means to shed light on why certain digital innovations were introduced in the first place, based on which considerations, and how technologies and digital innovations were constructed as solutions for similarly constructed problems. We may then start to understand which narratives and explanations drive technological innovation, as well as put our finger on the blind spots and gaps in these narratives. Is reliance on video-conferencing tools a consequence of economic constraints or security considerations, which outweigh concerns with the quality of dialogue between conflict parties? Are interventions to fight hate speech on social media easier to implement and measure, thus promising to demonstrate faster success than an arduous engagement with conflict stakeholders through training programs? When studying the effects of technologies on peacebuilding and linking them to technology design processes and the reasoning that underpins them, research will be able to provide critical insights into the political dynamics that underpin digital peacebuilding and its successes and failures (Hirblinger et al 2022).

## Re-humanizing digital peacebuilding

A post-digital perspective invites us to change our view on who or what *does* peacebuilding and who or what exercises power over peacebuilding dynamics and outcomes. The policy practice of digital peacebuilding seems obsessed with the usefulness and impact of specific “tools,” “apps,” or “platforms,”^9^ thus seemingly putting humans in the backseat of digital peacebuilding. In contrast, existing research has largely been concerned with different approaches, perspectives, paradigms, or agendas that shape peacebuilding (Carey, 2020; Richmond and Visoka, 2021), and has, to a considerable extent, structured around a debate around “liberal” peacebuilding and its alternatives (Mac Ginty and Richmond, 2013). Therefore, the debate has largely focused on human practices and agency. Liberal norms, international organizations, state sovereignty, or peace infrastructures, to mention only a few, are implicitly or explicitly taken as human-made. Likewise, “hybrid” or “post-liberal” peace was seen as a process or product of a variety of human actors and their practices—international and local—working in various degrees of friction and synergy toward peace. The existing peacebuilding scholarship is thus largely grounded in an enlightenment notion of human agency, understood as the capacity to make a difference based on free will and intentionality (Passoth and Rowland, 2015; Sayes, 2014).

The depiction of digital peacebuilding as something that is done by human agents who use digital tools creates an artificial dichotomy between humans and machines. The human claim to be distinct—and independent—from technology tends to emerge particularly often in moments of disenchantment, when the realization that humans and machines are entangled and interdependent may be rather unpleasant. This is well encapsulated in the trope of the cyborg (Haraway, 1991), which feeds on human fears of losing their humanness in the entanglements with machines; it gives rise to a disenchantment that asks us to other and externalize technology as distinctly nonhuman, and to reclaim and re-enact human agency. Where digital peacebuilding initiatives have limited success or undesirable effects, these tend to be attributed to a design failure or to the limited functionality, efficiency, or availability of tools—for instance, in terms of limited digital infrastructure and access, errors in prediction algorithms, or lacking data. An often-heard claim about online dialogue platforms, for example, is that they do not enable “meaningful” encounters. However, as Knox (2019: 167) has argued, the post-digital offers us the “recognition of the increasing sense that any analytic separation of “technology” and “humanness” fails to articulate our contemporary condition.” Moving beyond a naïve interest in the benefits or shortcomings of automation, the post-digital invites us to think about how technological and human agency are intertwined in complex relationships that mediate cognition and emotion (Jandrić et al., 2019: 174). This means acknowledging that machines have agency in the sense that they are often indispensable for human action and in that, they make a difference to the course of political processes and their outcomes, based on an understanding of agency as a distributed effect that emerges in human–machine networks (for a detailed discussion, refer Hirblinger, 2022; Hoijtink and Leese, 2019; Rammert, 2012).

While acknowledging the fact that tools may well have agency, the post-digital invites us to take a distinctly human-centered look at the socio-technical systems that jointly “do” peacebuilding. Through it, digitalization re-emerges first and foremost as something that is both shaped by, and shapes, humans—their practices, their way of life, their desires, and so on. Indeed, the post-digital conveys a need to extract human agency from the labyrinths of the digital, to both reassert and rearticulate the relevance of human agency—and to ask about the impact that digitalization has on the human condition (Jandrić et al., 2019: 173). Investigating peacebuilding outcomes, we may want to ask how critical choices about the purposes and underlying functions of technology-supported interventions are made in design processes or trace the decision-making processes that lead to deploying or not deploying a particular technology. Returning to the example of online dialogue platforms, re-humanizing peacebuilding would mean asking not only about the shortcomings of technologies but also about human needs, as well as human shortcomings—such as the unwillingness or inability of third parties to organize in-person meetings due to security, bureaucratic, or financial constraints—or mere personal preferences.

While we must account for digitalization as a distinct phenomenon, marked by the introduction of digital technologies into the social and political processes that may or may not build peace, it is crucial that we maintain enough intellectual distance so as not to lose focus of the fact that, ultimately, digital peacebuilding is affected by and affects humanity. Drawing on notions of agency that stress the distinctiveness of intentionality and freewill that underpin human agency (Emirbayer and Mische, 1998), we can attempt to re-humanize digital peacebuilding by asking about the role of human agents, as they intentionally break with certain schemas of action—or create new ones. Yet, this does not mean ignoring the interdependence of structure and agency (Giddens, 2013). For instance, the decision not to host a consultation remotely and rather make the effort to organize in-person dialogues that involves stakeholders with limited digital access or literacy, then emerges as a concrete choice that allows for more meaningful and in-depth encounters with the digitally less connected while potentially reducing the reach and efficiency of interventions. Similiary, professionals may decide not to employ off-the-shelf Natural Language Processing (NLP) and text analysis tools such as sentiment analysis or topic modeling, which are commonly used for marketing in the private sector because they create analysis that is meaningless for peacebuilding. Instead, they may choose to invest into the design of language models that map out arguments or narratives that could help to identify conflict party grievances and their understandings of conflict. Digital peacebuilding approaches and outcomes thus become perceivable as a result of human intention in the context of structural constraints—for instance, caused by technology design or a market logic that shapes innovation—and human agency returns into the spotlight of our attention because it is needed to alter them. The concerns that emerge as we re-humanize digital peacebuilding are as much as ethical as they are political, and they deserve further attention.

## Rhizomizing digital peacebuilding

Consequently, a final concern should be with the power relations that shape the dynamics and outcomes of digital peacebuilding. While the Twitter practice and policy discourse on digital peacebuilding demonstrates some interest in questions of “power” and “empowerment,”^10^ much of the existing academic research suggests that power relations in digital peacebuilding continue to be structured by what are conventionally viewed as the main building blocks of the international system, such as the state, capital, and the military (Richmond and Tellidis, 2020: 9). However, as Andersen et al. (2014) suggest, the post-digital asks us to be curious about how alternative constellations of actors may undermine, or cut, connections to conventional and new centers of power and create new alliances. An indication that this may be the case can be found in Tweets about the need to “adapt” to digital methods, seek new “partnerships,” or work in “networks” that extend to concerns with “health” and “climate” (Andersen et al., 2014). Once we accept that digital peacebuilding may best be understood in terms of socio-technical systems in which humans and machines have distributed agency, we see that presuming that these systems are composed of static entities or hierarchies would produce reifications that can result in methodological blind spots that reduce our capacity to study struggles over power. Building on Michel Foucault’s insight that “where there is power, there is resistance” (Foucault, 1990: 95), a critical analysis may want to avoid reproducing conventional ontologies—bottom-up versus top-down, local versus international or liberal, state versus civil society, and instead shed light on the relational dynamics of power and resistance that characterize digital peacebuilding.

To do so, a post-digital perspective should aim at a more fine-grained study of the dynamics of differentiation in socio-technical systems. I suggest that this may be achieved if we *rhizomize* peacebuilding—by adopting a perspective that puts the focus of our study not on presumably pre-given and stable arrangements but on the relational processes through which they are constructed, reconstructed, or deconstructed. The rhizome, prominently employed in a variety of studies of the digital, drawing on Gilles Deleuze and Félix Guattari, describes networks that ceaselessly establish and break connections between heterogenous elements in non-hierarchical, multiplous, and acentered ways (Gartler, n.d.). It has been employed in the study of Internet-based technologies such as the World Wide Web, social media, networking, and collaboration platforms (Buchanan, 2007; Burbules, 1997; Conley, 2005) and to transcend the binary differentiations of online and offline (Jones and Bennett, 2017). That said, a rhizome perspective would seek not to predetermine the entities that constitute digital peacebuilding nor the power relations between them. It directs our interest less toward the multiplous and heterogenous elements of socio-technical systems than toward the relationships between them and how they form and break, why they do so, and with what effects (Stephenson and Zanotti, 2017). If peacebuilding was ever conceivable as a closed and stable system composed of fixed elements, a rhizomatic perspective helps to demonstrate that the digitalization of peacebuilding enables arrangements that are more open, more adaptable, and more fluid. Rather than operating from the assumption that peacebuilding organizations and social media companies form fixed entities, the rhizome encourages us to look both beneath and beyond these units to ask about how socio-technical relations that matter for peacebuilding evolve and to explore how these arrangements break old connections and form new ones.

As a heuristic device, the rhizome compels us to look at how the making and unmaking of connections between its elements stratify power relations and how they prevent the consolidation of power in one place (Linstead and Thanem, 2007). It invites us to shed light on how conventionally powerful actors, such as international organizations and states, negotiate their influence with new actors, such as social media companies, tech companies, infrastructure providers, and PeaceTech organizations. In many conflict-affected contexts (and elsewhere), social media firms yield far-reaching influence over the information environment. To collect and use social media data for conflict prevention, peacebuilding actors such as the United Nations Development Program now advise building collaborations with “tech platforms” (UNDP, 2022). For instance, organizations that contribute to fighting hate speech commonly employ social media company–owned platforms such as Crowdtangle to identify problematic posts and will report them to Facebook’s content moderation teams. When training volunteers to identify and report hate speech, they orient their approach along the social media platforms’ Terms of Service. We may thus want to be concerned with how the granting of privileged access to social media data and analytics platforms might create new dependencies because it increases the power of social media companies in shaping conflict prevention efforts, coincidentally giving them a central role in mitigating the harmful effects of their own products and business models.

On the contrary, we may want to be curious about how socio-technical systems challenge the taken-for-granted centers of power, which contributes to the search for more desirable forms of peacebuilding. Indeed, some peacebuilding organizations have emerged as the most vocal critics of social media companies and their impact on armed conflict, having monitored and called out governmental efforts to censor content or black out the Internet. They provide knowledge and toolkits for conflict monitors, human rights defenders, and activists to strengthen their cyber resilience, protect against unwanted surveillance, and operate outside of conventional telecommunications infrastructures. Moreover, social movements and local start-up initiatives have demonstrated their ability to circumvent the influence of BIG-Tech through digital, open-source Do-It-Yourself solutions that enable new socio-technical constellations (Defy Hate Now, 2018).

To rhizomize peacebuilding also means breaking with singular narratives about digital peacebuilding and its effects. As Wilson and Nash (2011) argued in their study of digital media, “the post-digital represents a breakdown of the authoritarian structures which guide traditional narrative practice.” This means we will be left without a single compelling account of what digital peacebuilding is or how it represents itself. Yet, drawing on Deleuze, they argue that “our concern should be less with the ‘breakdown of these barriers’ but with ‘the reaction to the breakdown’ because ‘if the barriers are transgressed then the reaction of the author is to impose boundaries with greater force’” (Wilson and Nash, 2011). With that in mind, while taking the heterogenous, relational, and fluid nature of digital peacebuilding as a starting point, research should focus on attempts to regulate it, limit it, and dominate it in efforts to re-establish singular understandings of peace and peacebuilding. What comes to mind are attempts by authoritarian governments to regulate cyberspace or to censor peace initiatives or protest movements online (Jones, 2022), but likewise, efforts by international organizations to enforce singular notions of good Internet conduct and “cyber hygiene” (Stifel et al., 2022), or by peacekeeping missions to achieve post-facto discursive legitimation through “strategic communications” social media (Leib, 2023). A post-digital perspective invites us to study these frictional dynamics and how they shape peacebuilding outcomes.

## Conclusion

A post-digital research perspective on peacebuilding disavows any simple conclusion. This article suggests that we are neither at the beginning of a new era of digital peacebuilding nor at the end of it. Consequently, it is not time to declare a new turn to mobilize for a research agenda that would be fundamentally different from those that already exist. Instead, a post-digital lens asks us to engage with how human and machine relations evolve in socio-technical systems and how these shape dynamics and outcomes of peacebuilding here and now. It invites us to trace how in these rhizomatic arrangements, multiple relationships are constantly made and unmade, some knots are tightened while others are released, collaboration turns into friction, or seemingly tightly bound and institutionalized partnerships may start to leak with data. And certainly, the post-digital asks for research that takes stock of what grows in the new normal of digital peacebuilding in a rather sober way, moving beyond both fetishization and disillusionment by shedding light on the power relations that shape peacebuilding dynamics and outcomes while acknowledging that they are ultimately amenable to human agency. As we follow the relational dynamics through which humans and machines jointly build peace or fail to do so, the post-digital invites us to simultaneously stay with, and move beyond, the digital. This paves the way for a research perspective that demystifies the role of digitalization and enables critical scrutiny of its impact on contemporary and future peacebuilding.

## Supplemental Material

sj-xlsx-1-cac-10.1177_00108367231184727 – Supplemental material for When the digits don’t add up: Research strategies for post-digital peacebuilding Supplemental material, sj-xlsx-1-cac-10.1177_00108367231184727 for When the digits don’t add up: Research strategies for post-digital peacebuilding by Andreas T Hirblinger in Cooperation and Conflict

## Notes

1. The basis for this analysis contains all original Tweets with the terms “digital” and “peacebuilding” posted since the launch of Twitter in March 2006 until the end of 2021. A total of 944 tweets are included in the final data set. The tweets have been downloaded from Twitter via a research/developer account with the R-Studio AcademicTwitteR package, using the search terms “digital” and “peacebuilding.” Retweets and tweets with identical content were excluded. The corpus was analyzed with a combined manual and auto coding approach using GREP search, through which the author developed a set of keywords to identify claims related to the digital in peacebuilding and cluster them according to the seven intellectual maneuvers discussed in this article. The data can be accessed with the following link: https://doi.org/10.5281/zenodo.8058958.

2. This number is arbitrary to the degree to which further maneuvers could (and may) be added by other researchers, as they engage in the debate. Rather than providing a complete list, this article is more concern with demonstrating the viability of organizing research through intellectual maneuvers, and—as much as possible—documenting them based on the reading of existing research on the post-digital.

3. See tweet cluster 1.

4. See for instance, Remesh’s methodology for “mass online focus groups” (Bilich et al., 2019), or Build Up’s Phoenix social media listening tool for peacebuilders (Build Up, 2023).

5. See Tweet cluster 2.

6. See Tweet cluster 3.

7. See Tweet cluster 4.

8. See Tweet Cluster 5.

9. See Tweet Cluster 6.

10. See Tweet cluster 7.
